# The association of catestatin and endocan with the effects of cardiac shock wave therapy: Biomarker sub-study of the randomized, sham procedure-controlled trial

**DOI:** 10.3389/fcvm.2023.1004574

**Published:** 2023-02-23

**Authors:** Greta Burneikaitė, Evgeny Shkolnik, Roma Puronaitė, Gitana Zuozienė, Birutė Petrauskienė, Nerijus Misonis, Edita Kazėnaitė, Aleksandras Laucevičius, Fatima Smih, Philippe Rouet, Jelena Čelutkienė

**Affiliations:** ^1^Faculty of Medicine, Institute of Clinical Medicine, Vilnius University, Vilnius, Lithuania; ^2^Vilnius University Hospital Santaros Klinikos, Vilnius, Lithuania; ^3^LA Maison de la Mitochondrie (LAMMI), Obesity and Heart Failure: Molecular and Clinical Investigations, INSERM Occitanie, Toulouse, France; ^4^Section of Cardiovascular Medicine, Yale University School of Medicine, New Haven, CT, United States; ^5^Faculty of Mathematics and Informatics, Institute of Data Science and Digital Technologies, Vilnius University, Vilnius, Lithuania; ^6^Faculty of Medicine, Institute of Biomedical Sciences, Vilnius University, Vilnius, Lithuania; ^7^INI-CRCT-FCRIN, GREAT Networks, Toulouse, France; ^8^Spartacus-Biomed, Auterive, France; ^9^Centre of Innovative Medicine, Vilnius, Lithuania

**Keywords:** cardiac shock wave therapy, regenerative treatment, angiogenesis, stable angina, myocardial ischemia, biomarkers, catestatin, endocan

## Abstract

**Introduction:**

Cardiac shock-wave therapy (CSWT) is a non-invasive regenerative treatment method based on low-frequency ultrasound waves, which stimulate angiogenesis. Current data about the effects of revascularization procedures on angiogenesis biomarkers is limited. Recently, an association of catestatin and endocan with coronary collateral development was shown in several trials. In this study, we aimed to evaluate the impact of CSWT on the dynamics of catestatin and endocan levels and to assess their correlation with parameters of myocardial perfusion and function.

**Methods:**

Prospective, randomized, triple-blind, sham procedure-controlled study enrolled 72 adult subjects who complied with defined inclusion criteria (NCT02339454). We measured biomarkers in 48 patients with stable angina (24 patients of CSWT group, 24 patients of sham-procedure group). Additionally, patients were divided into responders and non-responders according to improvement in myocardial perfusion and/or contractility assessed by myocardial scintigraphy and dobutamine echocardiography (30 and 13 patients, respectively). The blood samples were collected at baseline, after the last treatment procedure (9th treatment week) and at 6-month follow-up to evaluate biomarkers concentration and stored at –80° until analysis. Serum catestatin and endocan levels were determined by commercially available ELISA kits.

**Results:**

Serum catestatin concentration significantly increased in all patients. While endocan levels significantly decreased in the responders sub-group. The increase in catestatin levels at 9th week and 6 months was positively associated with improvement in summed difference score (rho = 0.356, *p* = 0.028) and wall motion score, WMS (rho = 0.397, *p* = 0.009) at 6 months in the whole study population. Meanwhile, the decrease in endocan levels over 6 months was positively correlated with improvement in WMS at 3- and 6- months (*r* = 0.378, *p* = 0.015 and *r* = 0.311, *p* = 0.045, respectively). ROC analysis revealed that a change at 6 months in catestatin and endocan levels significantly predicted improvement in myocardial perfusion and contractile function with 68.9% sensitivity and 75.0% specificity (*p* = 0.039) and 51.7% sensitivity, and 91.7% specificity (*p* = 0.017), respectively. Baseline endocan concentration and its change at 6 months predicted response to CSWT with 68.8% sensitivity and 83.3% specificity (*p* = 0.039) and 81.3% sensitivity and 100% specificity (*p* < 0.0001), respectively.

**Conclusion:**

This study demonstrates the association of increase in catestatin and decrease in endocan levels with the improvement of myocardial perfusion and contractile function. The potential predictive value of catestatin and endocan dynamics for the response to regenerative therapy is shown.

## 1. Introduction

Coronary artery disease (CAD) remains a leading reason for adult mortality worldwide, responsible for 20% of deaths yearly in Europe (1), with stable angina being the most frequent clinical presentation. Significant advances in medical therapy and modern revascularization techniques using coronary artery bypass surgery or percutaneous intervention have markedly improved life expectancy and quality of life in patients with CAD. Traditional revascularization methods restore perfusion in large coronary arteries but remaining microvascular deficit may still induce adverse cardiac remodeling and functional deterioration. Angiogenic and regenerative treatment options potentially address this deficit by promoting progenitor cell differentiation to new myocardial cells including cardiomyocytes, endothelial or vascular smooth cells.

Cardiac shock wave therapy (CSWT) is one of the treatment methods for refractory angina pectoris that utilizes a non-invasive application of low-intensity shock waves directed to the target ischemic area. The mechanical stimulus of shock waves (SW) induces regenerative effects in ischemic tissue *via* localized stress on cell membranes that resemble shear stress, which leads to the release of angiogenic factors such as endothelial nitric oxide synthase (eNOS), vascular endothelial growth factor (VEGF), and proliferating cell antinuclear antigen (2–4). Expression of angiogenic growth factors modulates inflammatory response and enhances myocardial angiogenesis (5). Our previous review and meta-analysis that included 12 controlled clinical studies showed that in the most of them, Canadian Cardiovascular Society (CCS) angina class, angina frequency and nitroglycerine consumption decreased, and Seattle angina questionnaire (SAQ) scores improved (6). In addition, meta-analysis of 22 studies showed significant moderate improvement in exercise capacity (6). Yet, the molecular mechanism by which shock waves and shear stress promote neovascularization and improve cardiac function has not been entirely elucidated.

Angiogenesis is a process that forms new vessels from a the pre-existing vasculature in many physiological conditions. It is regulated by a complex interaction of pro- and antiangiogenic factors. Recently, catestatin and endocan were discovered as novel biomarkers involved in the vascular pathways (7, 8).

Catestatin is a 21-amino-acid-residue peptide derived from the neuroendocrine hormone chromogranin A (9, 10). Several *in vivo* studies showed that catestatin acted as an attenuator of the cardiac inflammation in hypertension, reduced sympathetic nerve activity and catecholamines secretion, decreased blood pressure and heart rate (11–14). There is considerable evidence that catestatin is a pleiotropic modulator against cardiovascular diseases, such as arterial hypertension (10), heart failure (15), and myocardial infarction (16).

Endocan, is a soluble proteoglycan of 50 kDa expressed by the vascular endothelium. It is considered to be a biomarker of the inflammatory process and endothelial cell activation (17, 18), since its increased levels have been shown not only in coronary artery disease (19), hypertension (18) and diabetes mellitus but also in the other inflammatory processes such as cancer (20). The expression of endocan is upregulated by pro-inflammatory molecules, including tumor necrosis factor-alpha (TNF-α), as well as pro-angiogenic molecules, including fibroblast growth factor 2 (FGF-2) and VEGF (17, 21–23).

Few single-arm studies revealed an increase in VEGF after CSWT (24–26). In a recent study, Martinez-Sanchez et al. (27) showed an increase in the number of endothelial progenitor cells (EPC) and concentration of angiopoietin-3, while the serum levels of interleukin 18 (IL-18) and transforming growth factor β decreased after CSWT.

It can be presumed that catestatin and endocan as new angiogenic markers might react to CSWT, a method of non-invasive regenerative therapy. Therefore, the present study aimed to evaluate the impact of CSWT on the dynamics of catestatin and endocan levels, compare them between the intervention and sham-procedure groups, and to assess their correlation with parameters of myocardial perfusion and contractile function.

## 2. Materials and methods

### 2.1. Study population

A prospective, randomized, triple-blind, sham-procedure controlled study was designed to assess the antianginal efficacy of CSWT on top of the standard medical therapy in patients with stable angina. The study protocol was created according to Consolidated Standards of Reporting Trials (CONSORT) statement recommendations for parallel group randomized trials (28). The study was conducted following the Good Clinical Practice and Declaration of Helsinki 2013. The main trial's design, methods, and results (NCT02339454) were described previously (29, 30).

Briefly, patients with angiographically confirmed coronary artery disease and exercise-induced angina associated with ST segment depression ≥1 mm on treadmill electrocardiogram (ECG) and symptoms not controlled by optimal medical treatment (OMT) were enrolled in the study. Exclusion criteria were angina at rest, acute coronary syndrome or planned coronary revascularization within 6 months, New York Heart Association (NYHA) heart failure class III-IV, thrombus in the left ventricle (LV), contraindications for exercise testing, ECG abnormalities at rest. Eligible subjects were assigned to the OMT + CSWT and the OMT + sham procedure study groups in a 1:1 ratio. Patients, investigators (clinicians and data assessors), and a statistician were blinded to treatment allocation.

### 2.2. CSWT treatment

All patients were maintained on stable doses of medications (31) for 4 weeks before the baseline evaluation and the entire study period. CSWT was performed using the Cardiospec device (Medispec Ltd., Germantown, Maryland, USA) using ECG R-wave gating. The target treatment area was determined by a cardiac ultrasound imaging (Vivid I; GE Healthcare, Horten, Norway). Treatment consisted of 9 sessions in total with 3 sessions per week on the 1^st^, 5^th^, and 9^th^ study weeks (30). The study population was evaluated at 3 and 6 months by clinical, exercise and imaging tests. The biomarkers were collected at baseline, 9^th^ week and 6 months.

### 2.3. Biomarkers tests

The biomarkers sub-study was conducted at Vilnius University Hospital Santaros klinikos (Vilnius, Lithuania) and was approved by Vilnius Regional Ethics Committee (Approval No. 158200-13-616-187).

Blood samples were collected at baseline, after the last treatment procedure at 9th treatment week and at 6-month follow-up visit (each time 5 ml). The samples were taken according to standard laboratory practice and centrifuged at 2,000 rpm for 20 min to collect the serum, then stored at –80°C for further biomarker analysis. Biomarkers measurements were performed at LA Maison de la Mitochondrie at INSERM institute (Toulouse, France).

Serum catestatin and endocan levels were determined by an enzyme-linked immunosorbent assay (ELISA) using commercially available diagnostic kits (Human catestatin ELISA Kit, cat. No: SL3027Hu, SunLong Biotech Co., LTD) and (Human Endocan/esm-1 ELISA Kit, cat. No: SL2210Hu, SunLong Biotech Co., LTD); intra-assay coefficient of variability (CV) <10%, inter-assay CV <12% for both. The analytical sensitivity of the catestatin commercial kit (lower detection limit for the test) is 5 pg/ml, with a linear range of 30–2,000 ng/mL; the sensitivity of endocan is 0.6 pg/ml, with a linear range of 3.3–200 ng/mL.

The quantification results were acquired using an ELISA plate reader (BL-ELIOC, Biobase Bioindustry (Shandong) Co., Ltd.) using the calibration curve to the kit manufacturer; the readings were performed at 450 nm wavelength.

### 2.4. Imaging parameters

The design, methods, and results of the imaging sub-study were described previously (32). Briefly, each sub-study patient underwent an exercise treadmill test, dobutamine stress echocardiography (DSE) and was assessed with Seattle Angina Questionnaire before the CSWT treatment and at 3- and 6-month follow-up, while echocardiography and single photon emission computed tomography (SPECT) was performed at baseline and 6 months.

Beta-blocking medications were discontinued for 48h, and other antianginal medications for 24h before stress tests as recommended in the Stress Echocardiography Expert Consensus Statement (33) and European Association of Nuclear Medicine procedural guidelines (34). Analysis of each DSE and SPECT images were performed by two independent observers blinded to the study data using the LV 17-segment model (35–37). Discordant assessments were jointly reviewed.

To evaluate the effect of CSWT on the dynamics of catestatin and endocan levels, the concentrations of biomarkers were compared between the OMT + CSWT and the OMT + sham procedure study groups. To assess the dynamics of biomarkers and their correlation with parameters of myocardial perfusion and function, patients were additionally divided into responders—those who had improvement in myocardial perfusion and/or contractility, and non-responders—those who did not show signs of improvement. Improvement in myocardial contractility was defined as the difference in wall motion score (WMS) before and after the study treatment at least by 3 points; improvement in perfusion was assessed as the difference in summed difference score (SDS) before and after the study treatment at least by 3 points. The patient was classified as a responder if ≥ 3 points change was demonstrated in either DSE or SPECT score, regardless of randomization group. For responders' analysis, we selected patients who had available DSE or SPECT tests at 6-month follow-up.

### 2.5. Statistical analysis

Baseline patients' characteristics were descriptively summarized: normally distributed continuous variables were expressed as mean value ± standard deviation (SD), non-normally distributed-as median [Q1-Q3], whereas categorical variables were expressed as absolute number (percentage). Paired parameters were tested for normal distribution with the Shapiro-Wilk test. Fisher exact test or Chi-square tests were used to compare categorical variables.

The difference between groups for variables with normal distribution was analyzed by using a parametric *t*-test, while for non-normally distributed variables, a non-parametric Mann-Whitney test was used. Wilcoxon signed rank test was used to compare paired data at baseline and follow-up, and repeated measures ANOVA was used to assess changes between groups to consider the time factor.

Pearson's or Spearman's correlation coefficients were computed to analyze the relationship between biomarkers and improvement in LV perfusion and function. A receiver operating characteristic (ROC) curve was plotted, along with computation of the area under the curve (AUC) and its 95% confidence interval. To reveal a potential prognostic value of catestatin and endocan for treatment response, a logistic regression model was created.

*P* < 0.05 (two-sided) were considered statistically significant. The overall effect of the CSWT was evaluated by comparing the average change of variable in the treatment group with the average change of variable in the sham procedure group.

Statistical analyses were performed with Statistica (version 13.3.0, TIBCO Software, Palo Alto, CA, USA) (38) and R version 4.1 for Windows (The R Foundation for Statistical Computing) (39).

## 3. Results

### 3.1. Baseline patients' characterisics

**Figure 1:**
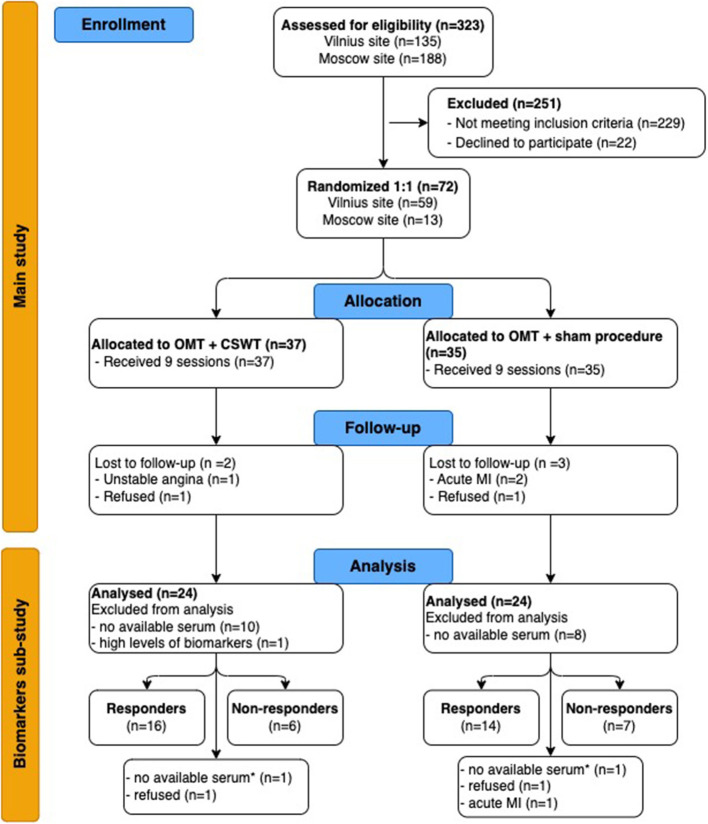
Flow chart of study (CONSORT 2010 Flow Diagram). CSWT, cardiac shock wave therapy; MI, myocardial infarction; OMT, optimal medical therapy. *-at 6-month follow up. For responders' analysis, we selected patients with available biomarkers levels and DSE or SPECT tests at 6-month follow-up: 22 and 21 patients of the OMT + CSWT and OMT + sham procedure group, respectively.

From June 2013 to December 2015, 72 patients were randomized (1:1) in the main study, 48 of them were included in the biomarkers sub-study: 24 patients entered the OMT+CSWT group and 24 patients were allocated to the OMT + sham procedure group (see Figure 1). One patient with extremely high biomarker levels (catestatin - 3588.2 pg/ml, endocan - 345.7 pg/ml) was excluded from the final analysis. No differences in clinical parameters, medical history, or treatment and no deviations from laboratory protocol were found in this patient compared with the entire group.

Baseline characteristics of the sub-study groups are presented in Table 1. The mean age of the patients was 68.5 ± 7.9 years with 75% men. Most patients (75%) had a multivessel disease and were not candidates for further revascularization due to the extent and severity of the disease or technical considerations. There were more males and patients with a history of myocardial infarction, as well as with a positive family history in the OMT + sham procedure group. Exercise capacity was moderately reduced in both study groups: total exercise duration in minutes was 5.6 (3.9; 7.6) and 5.4 (4; 7.1) in the OMT + sham procedure and in the OMT + CSWT group, respectively, *p* = 0.781.

**Table 1:** Baseline characteristics of the patients in sub-study groups.

**Variable**	**OMT + CSWT group (*n* = 24)**	**OMT + sham procedure group (*n* = 24)**	***P*-value**	**Responders (*n* = 30)**	**Non-responders (*n* = 13)**	***P* value**
**Demographic characteristics**						
Age, years	66.8 ±8	70.1 ± 7.6	0.126	76.7 ± 8.1	71.7 ± 3.7	0.031
Male sex, n (%)	15 (62.5)	21 (87.5)	0.048	22 (73.3)	11 (84.6)	0.351
**Cardiovascular risk factors**						
Hyperlipidemia, n (%)	24 (100)	24 (100)	-	30 (100)	13 (100)	-
Hypertension, n (%)	23 (95.8)	24 (100)	0.315	30 (100)	13 (100)	-
Diabetes, n (%)	5 (20.8)	7 (29.2)	0.506	5 (16.7)	5 (38.5)	0.125
Peripheral vascular disease, n (%)	7 (29.2)	9 (37.5)	0.546	9 (30)	6 (46.2)	0.312
Current or previous smoker, n (%)	4 (16.7)	9 (37.5)	0.109	8 (26.7)	3 (12.5)	0.311
Positive family history for CVD, n (%)	7 (29.2)	16 (66.7)	0.010	7 (53.8)	13 (43.3)	0.532
**Medical history**						
Previous myocardial infarction, n (%)	11 (45.8)	19 (79.2)	0.018	18 (60)	8 (61.5)	0.927
Previous PCI, n (%)	13 (54.2)	12 (50)	0.773	15 (50)	5 (38.5)	0.493
Previous CABG, n (%)	15 (62.5)	16 (66.7)	0.763	19 (63.3)	7 (53.8)	0.563
Three-vessel disease, n (%)	18 (75.0)	18 (75.0)	0.214	25 (83.3)	9 (69.2)	0.303
**Clinical parameters**						
Body mass index, kg/m^2^	30.2 ± 3.6	30.0 ± 4.0	0.888	29.5 ± 3.5	31.7 ± 4.2	0.077
Angina episodes/week	5 (3; 14)	7 (3.8; 14.3)	0.950	10.0 (3.0; 15.0)	4.0 (3.0; 12.0)	0.232
Systolic blood pressure, mmHg	135.0 ± 22.1	141.0 ± 21.6	0.351	131.0 ± 20.7	145.9 ± 20.8	0.036
Diastolic blood pressure, mmHg	80 ± 10.1	76.6 ± 10.8	0.397	77.7 ± 10.9	75.6 ± 7.8	0.531
LVEF (echocardiographic), %	54.9 ± 9.7	56.9 ± 7.1	0.645	56.6 ± 8.5	56.8 ± 7.0	0.493
**Angina CCS class**						
II, n (%)	8 (33.3)	8 (33.3)	1	11 (36.7)	3 (23.1)	0.388
III, n (%)	16 (66.7)	16 (66.7)		19 (63.3)	10 (76.9)	
SAQ score total, mean	68.3 ± 12.4	66.7 ± 13.1	0.677	66.9 ± 11.7	70.1 ±11.7	0.576
**Medical treatment**						
ACE inhibitors/ARB, n (%)	24 (100)	24 (100)	-	30 (100)	13 (100)	-
Beta-blocker, n (%)	23 (95.8)	22 (91.7)	0.562	28 (93.3)	12 (92.3)	0.907
Long-acting nitrates, n (%)	14 (58.3)	11 (45.8)	0.391	15 (50.0)	7 (53.8)	0.821
Calcium channel blocker, n (%)	13 (54.2)	11 (45.8)	0.565	16 (53.3)	7 (53.8)	0.976
Another antianginals*, n (%)	15 (62.5)	15 (62.5)	1	20 (66.7)	6 (46.2)	0.212
Statins, n (%)	24 (100)	24 (100)	-	30 (100)	13 (100)	-
Mean dose of atorvastatin, mg	37.1 ± 12.3	39.6 ± 16.3	0.552	40.7 ± 13.9	32.3 ± 9.3	0.143
Antiplatelets, n (%)	24 (100)	24 (100)	-	30 (100)	13 (100)	-
**ECG exercise test**						
Exercise duration, min	5.4 (4.0; 7.1)	5.6 (3.9; 7.6)	0.781	5.6 (4.2; 7.4)	4.2 (3.5; 6.6)	0.125
**Dobutamine stress echocardiography**						
Wall motion score at stress	25.5 (21.0; 31.3)	25.5 (23.0; 28.5)	0.434	25.5 (22.0; 30.0)	23.0 (20.0; 32.0)	0.757
Δ Wall motion score	3.3 ± 3.7	3.0 ± 4.7	0.687	4.4 ± 3.5	1.9 ± 4.0	0.040
**Single photon emission computed tomography**						
Summed difference score	6.0 (4.0; 8.5)	4.5 (3.0; 9.5)	0.868	6.0 (4.0; 10.0)	4.0 (3.0; 5.0)	0.014

ACE, angiotensin-converting enzyme; ARB, angiotensin receptor blocker; CABG, coronary artery bypass grafting; CCS, Canadian cardiovascular society; CSWT, cardiac shock wave therapy; CVD, cardiovascular disease; ECG, electrocardiogram; LVEF, Left ventricular ejection fraction; PCI, percutaneous intervention; SAQ, seattle angina questionnaire, OMT, optimal medical therapy; *other antianginals include trimetazidine and ranolazine. Δ Wall motion score = WMS at stress-WMS at rest. *P* < 0.05 considered as significant.

The main study showed neutral effects of the addition of CSWT on exercise tolerance and symptoms in patients with stable angina (29). However, imaging sub-study results revealed that CSWT effectively improved myocardial perfusion and left ventricle (LV) function during stress (32). An example of the effects of cardiac shock wave therapy on top of optimal medical treatment is shown in Supplementary Figure S1.

Complete data of the catestatin and endocan levels and DSE were available for 22 patients of the OMT + CSWT group and 23 patients of the OMT + sham procedure at 3-months follow-up, and 22 patients of each group at 6 months follow-up. Meanwhile, complete data of the catestatin and endocan levels and perfusion imaging by SPECT were available in 20 and 21 patients of the OMT + CSWT and OMT + sham procedure group, respectively. Out of 43 patients, 30 (69.8 %) positively responded to treatment with improvement in perfusion and/or contraction scores ≥ 3 points. Responders were significantly younger and had higher systolic blood pressure (Table 1). The trend of more frequent history of type 2 diabetes and peripheral artery disease, exercise duration shorter by 1.5 min and fewer angina episodes were observed in the non-responders' sub-group. Also, there were fewer revascularization procedures and less amount of inducible ischemia among non-responders (SDS 4.0 [3.0; 5.0] vs. 4.0 [3.0; 5.0], *p* = 0.014 and Δ WMS [WMS at stress-WMS at rest] 1.9 ± 4.0 vs. 4.4 ± 3.5, *p* = 0.04). Detailed characteristics of imaging parameters are presented in Supplementary Table S1.

### 3.2. Dynamics of catestatin during CSWT

**Figure 2:**
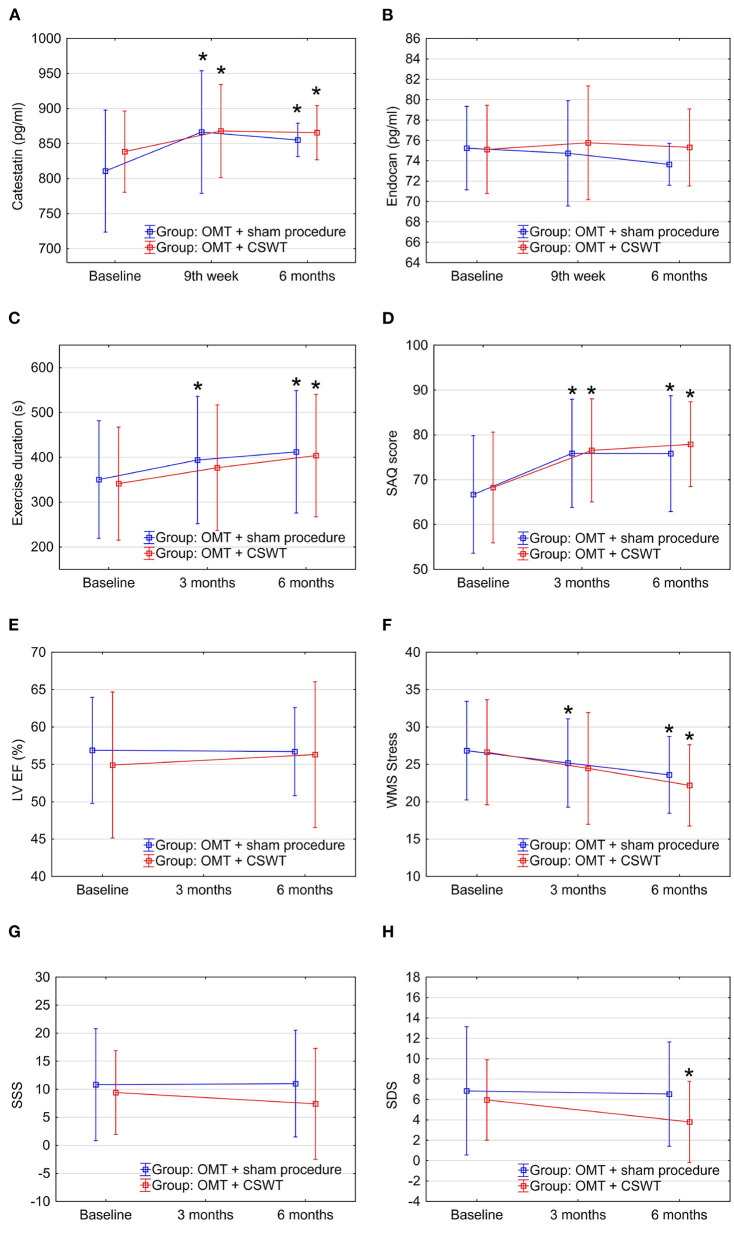
Dynamics of biomarkers, exercise, and imaging parameters in the sub-study groups: OMT + CSWT vs. OMT + sham procedure. Data are shown as mean ± SD. CSWT, Cardiac shock wave therapy; LV EF, left ventricle ejection fraction; OMT, optimal medical therapy; SAQ, seattle angina questionnaire; SDS, summed difference score; SSS, summed stress score; WMS, wall motion score. *P < 0.05, comparison of follow up to baseline in the group.

During the study, significant increase in serum levels of catestatin was found at 9^th^ week and maintained at 6-month follow-up in OMT + CSWT as well as in the control group (Figure 2A and Table 2). The absolute catestatin levels and their change did not differ significantly between the groups at all three-time points (Figure 2A and Table 2). The observed changes in catestatin level were paralleled by trends of increase in exercise duration (Figure 2C), improvement in SAQ score (Figure 2D) and a decrease in the amount of inducible ischemia expressed by WMS at stress (Figure 2F), SDS (Figure 2H) and summed stress score (SSS) (Figure 2G).

**Table 2:** Dynamics of biomarkers levels.

	**Catestatin, pg/ml**			**Endocan, pg/ml**		
	**Baseline**	**9th week**	**6-month**	**Baseline**	**9th week**	**6-month**
OMT + CSWT group (*n* = 24)	839.4 (809.7; 862.6)	864.5 (818.7; 903.2)^*^	856.6 (843.1; 880.8)^*^	74.8 (72.5; 76.4)	75.8 (72.0; 79.5)	75.0 (72.5; 76.9)
OMT + sham procedure group (*n* = 24)	814.0 (752.0; 854.8)	861.5 (826.8; 878.9)^*^	854.6 (842.7; 866.1)^*^	75.3 (71.6; 77.3)	75.5 (72.5; 77.4)	73.7 (72.2; 74.9)
Responders (*n* = 30)	839.6 (806.6; 860.0)	863.7 (801.2; 912.1)^*^	848.2 (835.2; 878.1)	75.8 (71.9; 78.7)	76.0 (72.2; 77.9)	73.5 (72.1; 75.2)^*^
Non-responders (*n* = 13)	798.6 (744.7;834.6)	856.0 (818.7; 870.1)^*^	858.7 (847.0; 881.4)^*^	73.7 (71.1; 75.4)	74.0 (71.8; 79.0)	74.7 (72.5; 76.3)

CSWT, cardiac shock wave therapy; OMT, optimal medical therapy.

* *P* < 0.05, comparison of follow-up to baseline in the group.

**Figure 3:**
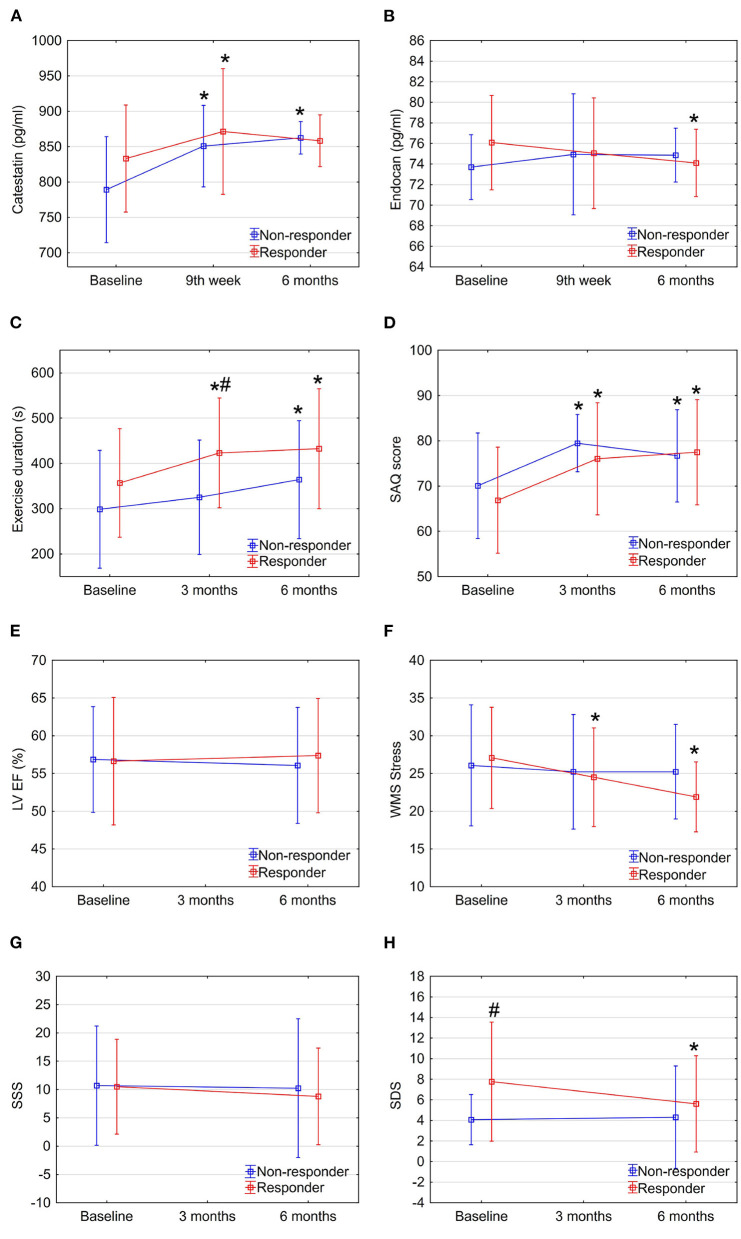
Dynamics of biomarkers, exercise, and imaging parameters in study sub-groups: Responders vs. non-responders. Data are shown as mean ± SD. CSWT, cardiac shock wave therapy; LV EF, left ventricle ejection fraction; OMT, optimal medical therapy; SAQ, seattle angina questionnaire; SDS, summed difference score; WMS, wall motion score. *P < 0.05, comparison of follow up to baseline in the group, ^#^*P* < 0.05, comparison between responders and non-responders. Repeated measures ANOVA for exercise duration (part C) between responders and non-responders *P* < 0.05.

Interestingly, when analyzing responders vs. non-responders, though catestatin levels also increased in both sub-groups (Figure 3A and Table 2), responders had increased catestatin levels and reduced endocan levels at 6 month. A tendency for higher levels of catestatin at baseline and 9^th^ week was observed in responders. Dynamics of catestatin levels in responders were associated with significantly increased exercise capacity within the sub-group and compared to non-responders (Figure 3C), increase in SAQ score (Figure 3D), LVEF (Figure 3E), improvement in myocardial perfusion (Figure 3G), and contractility during stress (Figure 3F).

### 3.3. Dynamics of endocan during CSWT

In contrast, endocan levels have shown a trend of decrease in the OMT + sham procedure group but remained relatively stable in the intervention group (Figure 2B and Table 2). At the same time, a slight improvement in LVEF (Figure 2E) and reduction of stress myocardial ischemia expressed by SDS were observed in the intervention group (Figure 2H).

Dynamics of endocan levels showed opposite directions in responders' and non-responders' sub-groups: it significantly decreased in responders while increasing in non-responders (Figure 3B and Table 2). The dynamics of endocan in the responders' sub-group paralleled changes in myocardial perfusion (Figures 3G, H) and contractility (Figure 3F).

Percentage change of catestatin levels showed an increase in all groups, but the increase quantitatively was lower in the intervention and responders' sub-groups (Supplementary Figures S2A, B). Meanwhile, the percentage change of endocan levels revealed contrary directions, decreasing in the control sub-group and in the responders' sub-group, while increasing in the intervention and non-responder's subgroups (Supplementary Figures 2C, D).

### 3.4. Association of catestatin and endocan levels with clinical and imaging parameters

**Figure 4:**
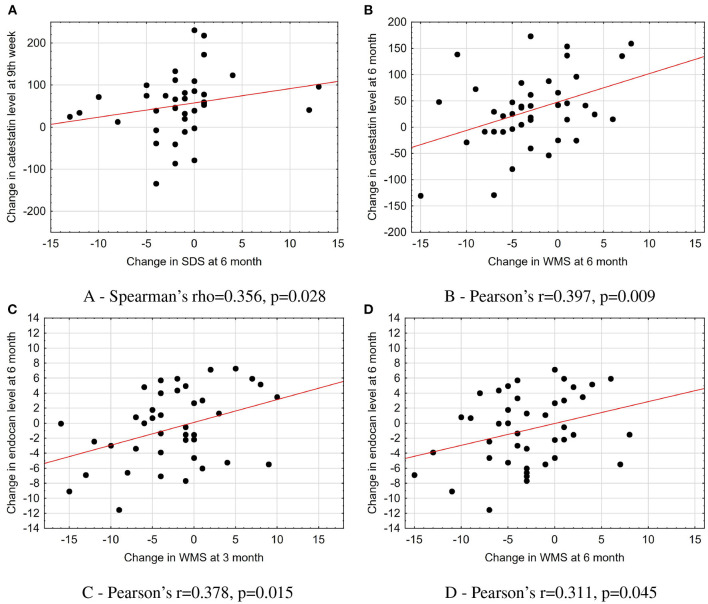
Association of changes in catestatin and endocan levels with parameters of imaging tests in the total population. **(A)** Spearman's rho = 0.356, *p* = 0.028. **(B)** Pearson's *r* = 0.397, *p* = 0.009. **(C)** Pearson's *r* = 0.378, *p* = 0.015. **(D)** Pearson's *r* = 0.311, *p* = 0.045. The scatterplots with a line fit using linear regression illustrate the associations between dynamics in biomarkers levels and changes in imaging test parameters. SDS, summed difference score; WMS, wall motion score.

The increase in catestatin levels at 9^th^ week and 6 months was positively associated with the reduction of the amount of stress-induced ischemia (expressed as changes in WMS and SDS) in the whole study population (Figures 4A, B). Meanwhile, the decrease in endocan levels over 6 months was positively correlated with the improvement in LV contractile function at 3 and 6 months (Figures 4C, D).

We found that endocan level at baseline was negatively associated with a tricuspid annular plane systolic excursion (Spearman's rho = –0.352, *p* = 0.015) in the total population. In the OMT + CSWT group, a moderate correlation was found between catestatin concentration and the amount of reversible ischemia (expressed as SDS score) at baseline (Pearson's *r* = 0.599, *p* = 0.002). Moreover, among responders, the increase in catestatin levels was moderately correlated with the improvement in exercise capacity (Δ exercise time, Pearson's *r* = 0.457, *p* = 0.043). While changes in endocan and catestatin did not show any correlations at all time points (Spearman rho was < 0.7, *p* > 0.05 for all comparisons).

### 3.5. The value of catestatin and endocan to predict a treatment response

**Figure 5:**
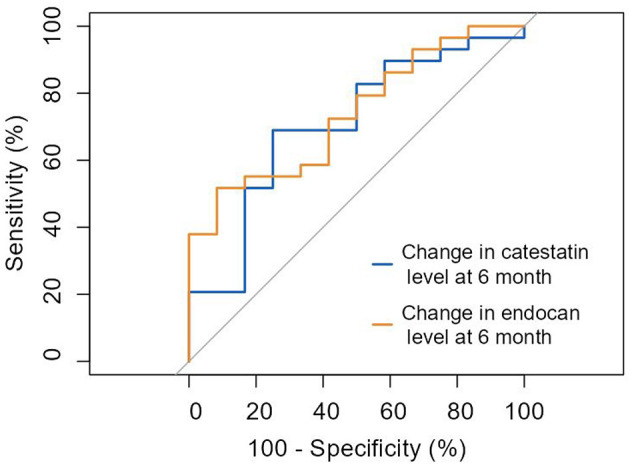
ROC curve: prediction of improvement in myocardial perfusion and/or contractile function by ≥3 points by the change of catestatin and endocan levels at 6 months. Change in catestatin level at 6-month (AUC = 0.707 [0.53-0.88], *p* = 0.039). Change in endocan level at 6-month (AUC = 0.739 [0.58-0.90], *p* = 0.017).

According to receiver operating characteristic (ROC) analysis, a change at 6 months in catestatin and endocan levels significantly predicted improvement in myocardial perfusion and contractile function with 68.9% sensitivity and 75.0% specificity (AUC = 0.707; 95% CI: 0.53–0.88; cut-off value 40.4 pg/ml, *p* = 0.039) and 51.7% sensitivity, and 91.7% specificity (AUC = 0.739; 95% CI: 0.58–0.90; cut-off value of 2.45 pg/ml, *p* = 0.017), respectively (Figure 5).

**Figure 6:**
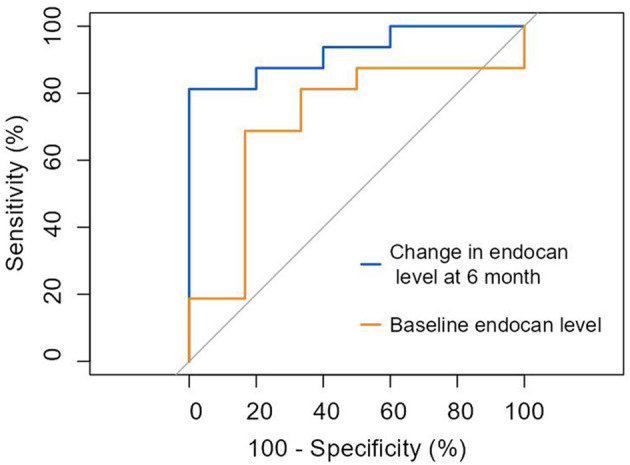
ROC curve: prediction of response to cardiac shock wave therapy according to improvement in myocardial perfusion and/or contractile function by ≥3 points by endocan levels. Baseline endocan level (AUC = 0.719 [0.51–1.01], *p* = 0.039). Change in endocan level at 6-month (AUC = 0.925 [0.83–1.05], *p* < 0.0001).

Additionally, we assessed the ability of the two biomarkers to predict response to treatment in the OMT+ CSWT group only. Baseline endocan concentration was associated with a treatment response with 68.8% sensitivity and 83.3% specificity (AUC 0.719; 95% CI: 0.51–1.01; cut-off value of 74.28 pg/ml, *p* = 0.039). Furthermore, the change in endocan levels at 6-month follow up predicted responders with 81.3% sensitivity and 100% specificity (AUC = 0.925; 95% CI: 0.83–1.05; *p* < 0.0001; Figure 6).

## 4. Discussion

This sub-study of the randomized CSWT trial assessed the changes of two biomarkers of angiogenesis and their relationship to the parameters of myocardial perfusion and contractile function. First, we found that changes in catestatin and endocan levels were associated with the decreased amount of stress-induced ischemia. Second, the change at 6 months in catestatin and endocan levels significantly predicted improvement in myocardial perfusion and contractile function. Moreover, we showed that endocan is a potential predictor for the response to CSWT.

Experimental studies have showed that the application of SW might induce the release of endothelial nitric oxide synthase (eNOS) in endothelial cells, which cause vasodilation and stimulates release of VEGF, mobilization of EPC in ischemic zones, leading to angiogenesis and the emergence of new capillaries (40–42). In addition, Nishida et al. demonstrated improvement of LV systolic function, wall thickening fraction and regional myocardial blood flow (42). Moreover, Fu et al. demonstrated that CSWT markedly increased the amount of endothelial progenitor cells and EPC homing-related chemokines in LV ischemic area, enhanced angiogenesis, reduced inflammatory response, oxidative stress, cellular apoptosis, and fibrotic changes in LV myocardium (4). These effects may contribute to the improvement in LV function and reverse remodeling.

There is a lack of data on molecular mechanisms of shock wave-induced angiogenesis in humans. Some results on CSWT impact on biomarkers are available from single-arm studies. Cai et al. observed a significant increase in the number of circulating EPC, mediated by VEGF and IL-8 secretion (26). Martinez-Sanchez et al. showed an increase in the number of EPC and concentration of angiopoietin-3, while the serum levels of IL-18 and transforming growth factor β decreased after CSWT therapy (27). Moreover, increased LxA4, VEGF and IL-1β levels were observed in patients with reduced myocardial ischemia after treatment (27).

*Angiogenesis* is a natural endogenous mechanism leading to vascular collateralization to preserve myocardial viability during ischemia and is regulated by a complex interaction of pro- and antiangiogenic factors, signaling cascades and cellular processes, including cell migration, proliferation and tubulogenesis (43). Recently, catestatin and endocan were discovered as novel angiogenic biomarkers. Catestatin contributes to the regulation of inflammation by modulating multiple immune cell functions. It has been shown that catestatin induces monocyte and mast cell migration, degranulation, and promotes the production of inflammatory chemokines (44, 45). Furthermore, monocytes and macrophages secrete growth factors and have the ability to differentiate into endothelium-like cells, thus contributing to angiogenesis (46–48). Moreover, a mouse model showed that catestatin reduces monocyte and macrophage infiltration in the heart (11) reducing inflammation (49). The proangiogenic action of catestatin depends on a basic fibroblast growth factor released from the endothelial cells (7). The study *in vitro* showed that catestatin induced migration and proliferation of endothelial cells and employed capillary tube formation *via* G protein and mitogen-activated protein kinase. The increase in catestatin levels is potentially associated with enhance in collateral network, which might explain the beneficial effects of CSWT on myocardial perfusion and contractile function in our study (49).

Endocan is expressed and secreted by endothelial cells, and its secretion is regulated by inflammatory cytokines (interleukin-1β, tumor necrosis factor-α, interferon-γ) (50) and proangiogenic factors (VEGF-A and VEGF-C) (51). Rocha et al. (8) demonstrated *in vivo* that endocan increases VEGF-A bioavailability and promotes VEGF-A signaling, leading to vascular permeability and vascular outgrowth. Moreover, endocan promotes the expression of inter-cellular adhesion molecule-1 (ICAM-1) and releases IL-8 and monocyte chemoattractant protein 1 (52). In this way, endocan acts on components that provide the essential substrate for recruitment, adhesion, and migration of leukocytes across the activated vascular endothelium, leading to angiogenesis.

The present study assesses the associations between dynamics of biomarkers and broad-spectrum parameters, including clinical, exercise and imaging, as a response to regenerative treatment. We studied the serum levels of catestatin and endocan at several time points—baseline, at 9-week and 6-month follow-up. The median baseline catestatin and endocan levels in the total study population were 424.6 (797.3; 859.2) pg/ml and 75.2 (71.9; 77.1) pg/ml, respectively. In recent reviews, high variability of catestatin (400–21,400 pg/ml) and endocan (260 - 1200 pg/ml) concentrations were observed in healthy controls when comparing different studies (53, 54). These variabilities might be due to differences in the health status of evaluated populations, serum or plasma sample used and different quantification methods.

Liu et al. showed that the levels of catestatin in patients with stable angina, acute coronary syndrome (15), and with slow coronary flow (55) tended to be higher than those seen in healthy controls. Furthermore, Xu et al. revealed that catestatin levels were significantly higher in patients with chronic total occlusion who underwent percutaneous coronary intervention (1.97 ± 1.01 ng/ml) compared with patients with chest pain and without angiographically significant stenoses (1.36 ± 0.97 ng/ml, *p* = 0.009) (56). Contrary, Chen et al. demonstrated that serum catestatin levels were lower in patients with significant CAD compared to healthy subjects and were inversely correlated with disease severity (57). It is challenging to interpret these discrepant findings, especially when associations between catestatin and anatomical abnormalities were not linked to the disease symptoms (chest pain) or the dynamics of biomarkers.

Several studies showed that endocan was significantly and consistently elevated in patients with stable angina (58, 59) including microvascular angina (60), compared to healthy controls. In addition, endocan values did not differ between patients with microvascular angina and patients with obstructive CAD (60), possibly reflecting dysfunction of the endothelium in both conditions. Kup et al. showed that endocan levels were significantly higher in patients with in-stent restenosis compared to patients with stable angina (61).

In our study, the baseline amount of stress-inducible myocardial ischemia did not differ significantly between the intervention and sham-procedure sub-groups. However, a significantly higher degree of ischemia expressed by SDS, along with higher endocan levels at baseline was found in the responders' sub-group, compared with the non-responders' sub-group. Meanwhile, after the treatment, both the endocan level and the magnitude of inducible ischemia decreased significantly in the responder's group. Therefore, it can be presumed that elevated baseline endocan levels are pathogenetically associated with inducible ischemia, and the decline in the levels of this endothelial proteoglycan reflects normalization of myocardial perfusion and subsequently contractile function.

Observing an improvement in myocardial perfusion and contractile function simultaneously with a decrease in the level of endocan, it can be assumed that the mechanism involved might be an increase in collateral network. Previously it was shown that higher endocan levels are associated with poor collaterals (62). At the same time, in patients with at least one chronic occlusion, Emet et al. demonstrated that serum endocan levels were significantly higher in the well-developed collateral group compared to the poorly developed collateral group (63). In the studies of catestatin, higher levels were associated with good coronary collateral development (56) and were unrelated to VEGF. While other studies analyzed biomarkers in observational cohorts, we had the opportunity of studying the dynamics of new biomarkers in a randomized clinical trial, allowing us to examine the interaction between baseline and follow-up biomarker levels and the efficacy of regenerative therapy. We found that decrease in endocan levels was associated with the reduction of the amount of stress-induced ischemia. Our study findings agree with those studies which evaluated the impact of coronary artery bypass grafting on the dynamics of endocan levels and showed a trend of decreasing after cardiac surgery (64–66).

The ROC analysis revealed that changes in catestatin and endocan levels reliably predicted improvement of myocardial perfusion and contractile function in the total study population. Furthermore, among patients who received true regenerative treatment on top of optimal medical therapy, baseline endocan levels and its decline at 6 months were significant predictors for CSWT response with high specificity. These findings raise the hypothesis that endocan could be used as a marker of response to revascularization and regenerative therapy. This should be investigated in a more extensive, controlled study.

## 5. Limitations

This is a *post-hoc* analysis of a prospective study with a relatively small sample. The prolonged storage of the samples could have an influence on the accuracy of measurements. Larger studies are required to confirm the association of these two new biomarkers with the application of regenerative therapy and to identify valid cut-off levels of catestatin and endocan to predict the response to treatment.

## 6. Conclusions

This study demonstrates the association of an increase in catestatin levels and a decrease in endocan levels with an improvement of myocardial perfusion and contractile function in the randomized cardiac shock wave therapy study. The potential predictive value of the dynamics of catestatin and endocan for the response to regenerative therapy is shown. Further studies are required to investigate the possibility of using endocan and catestatin for the selection of revascularization interventions (11).

## Data availability statement

The raw data supporting the conclusions of this article will be made available by the authors, without undue reservation.

## Ethics statement

The studies involving human participants were reviewed and approved by Vilnius Regional Ethics Committee. The patients/participants provided their written informed consent to participate in this study.

## Author contributions

GB, PR, and JČ designed the writing framework. GB and RP performed the statistical analysis and drew the pictures. GB wrote the first draft of the manuscript. ES, RP, GZ, BP, NM, EK, AL, FS, PR, and JČ revised and refined the manuscript. All authors have contributed, read, and approved the final manuscript.
