# Supplementary material for: The association of catestatin and endocan with the effects of cardiac shock wave therapy: Biomarker sub-study of the randomized, sham procedure-controlled trial
Source: Front Cardiovasc Med. 2023 Feb 23;10:1004574. doi: 10.3389/fcvm.2023.1004574 (PMC9996196; doi:10.3389/fcvm.2023.1004574)
Supplement: Supplementary file 1 [file Data_Sheet_1.PDF]

Supplementary Material

1 SUPPLEMENTARY DATA

1.1 Supplementary Tables and Figures

Table S1. Characteristics of the imaging tests in the sub-study groups.

	OMT + CSWT group			OMT + sham procedure group			Responders			Non-responders		
	Baseline	3-month	6-month	Baseline	3-month	6-month	Baseline	3-month	6-month	Baseline	3-month	6-month
Dobutamine stress echocardiography												
WMS at rest	19.5 (17.0; 25.5)	19.5 (17.3; 29.0)	19.0 (17.0; 24.3)	22.5 (17.0; 27.0)	23.0 (18.0; 27.5)	23.5 (17.3; 25.8)	20.5 (17.0; 24.0)	20.5 (17.0; 29.5)	21.0 (17.0; 27.0)	22.0 (19.0; 30.0)	20.0 (17.0; 27.0)	19.0 (17.0; 25.0)
WMS at stress	25.5 (21.0; 31.3)	21.0 (19.3; 29.0)	19.5 (17.3; 27.0)*	25.5 (23.0; 28.5)	24.0 (20.5; 29.5)*	22.0 (20.0; 26.8)*	25.5 (22.0; 30.0)	22.5 (19.0; 30.0)*	20.5 (18.0; 26.0)*	23.0 (20.0; 32.0)	25.0 (20.0; 27.0)	25.0 (19.0; 28.0)
LVEF at rest, %	46.5 ± 10.7	47.3 ± 11	51.1 ± 7.9*	48.3 ± 8.8	47.6 ± 9.3	48.0 ± 8.7	48.5 ± 8.3	47.3 ± 9.8	49.5 ± 7.8	48.1 ± 11.6	48.9 ± 10.8	50.8 ± 9.1
LVEF at stress, %	50.4 ± 11.7	54.1 ± 12.1	58.4 ± 7.8*	50.6 ± 10.8*	54.3 ± 10.8	53.6 ± 9.7	51 ± 10.6	54.5 ± 10.9*	57 ± 8.9*	51.2 ± 13.6	55 ± 11.6	53.9 ± 9.6
Single photon emission computed tomography												
SDS	6.0 (4.0; 8.5)	-	3.5 (0.0; 5.0)*	4.5 (3.0; 9.5)	-	5.0 (3.0; 9.0)	6.0 (4.0; 10.0)#	-	5.0 (2.0; 8.5)*	4.0 (3.0; 5.0)	-	3.0 (2.0; 5.0)
SSS	9.5 (5.5; 12.0)	-	5.0 (1.0; 9.0)	7.5 (3.5; 15.5)	-	8.0 (3.0; 18.0)	9.5 (5.0; 13.0)	-	5.5 (2.5; 13.0)	7.0 (4.0; 15.0)	-	6.0 (2.0; 14.0)
SRS	1.5 (0; 5.0)	-	0.0 (0.0;5.0)	1.5 (0; 6.0)	-	2.0 (0.0; 6.0)	1.5 (0; 4.0)	-	0.5 (0.0; 5.0)	1.0 (0; 9.0)	-	0.0 (0.0; 6.0)

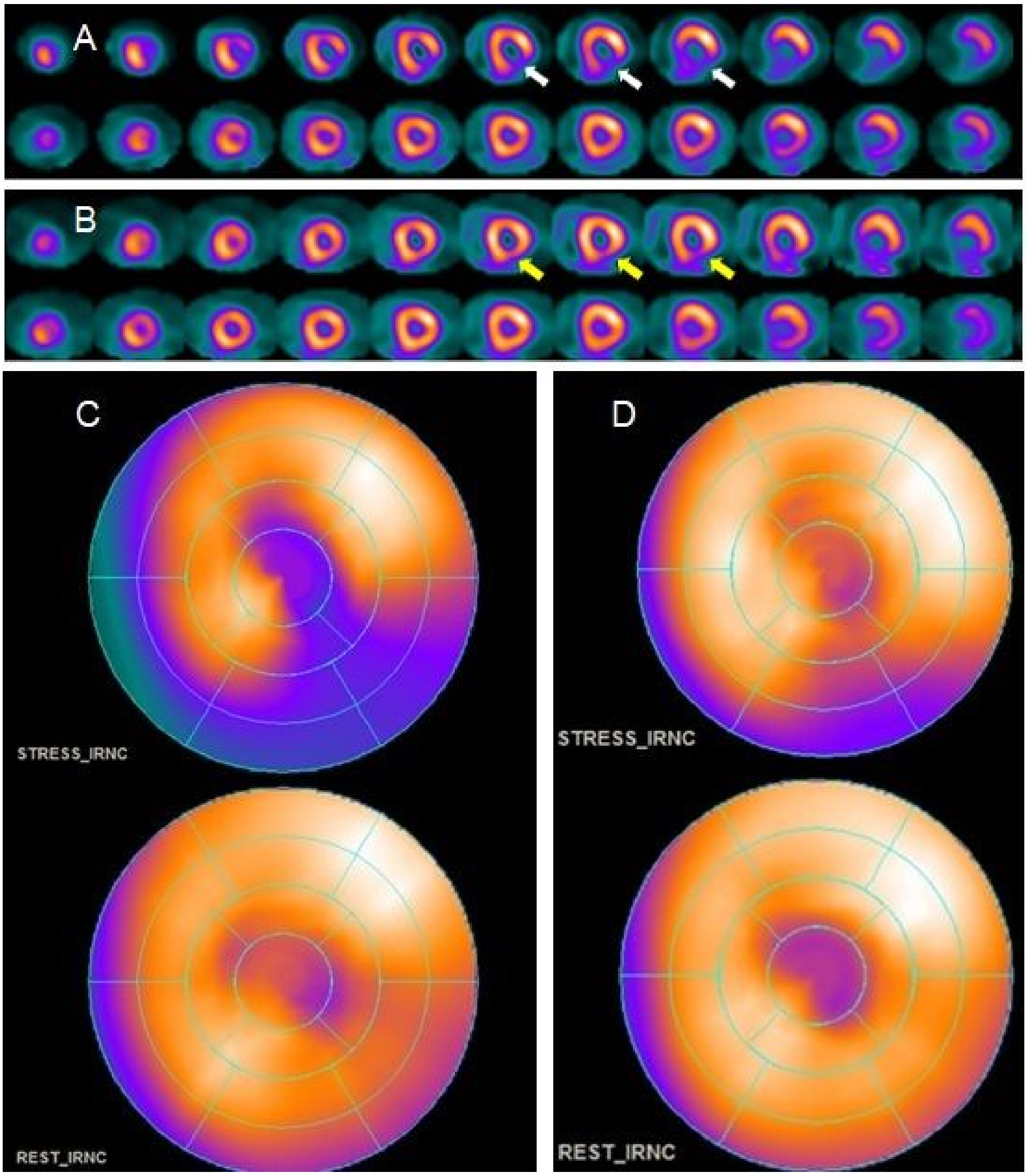

Figure S1. Tc-99m Sestamibi single day myocardial perfusion SPECT images at baseline and follow-up Tc-99m Sestamibi single day myocardial perfusion SPECT images of the 64 years old female patient with a significant three-vessel disease after CABG: right coronary artery (RCA) occlusion, left anterior descending artery (LAD) with diffuse lesions (70-90%), left circumflex artery (LCX) with stenoses (60-80%). Arterial graft (LIMA into LAD 7s) and venous graft into ROMI are without significant lesions, while venous grafts into ROMII and RIP are occluded.

A – The patient's baseline stress/ rest myocardial perfusion SPECT images showing partially reversible perfusion defect in the inferior, posterior, lateral and partially anterior wall and apex (marked with white arrows). The top rows (short axis from apex to base) show stress images, and the bottom rows show rest images.

B - SPECT sestamibi MPI at 6-month follow-up after treatment with cardiac shock wave therapy (MPI image format same as A). The amount of ischemia was significantly reduced (marked with yellow arrows).

C - Polar maps (at baseline): summed stress score (SSS) – 17, summed rest score (SRS) – 1, summed difference score (SDS) – 16, estimated the extent of total ischemia to be 24% of the left ventricular myocardium. The left ventricular ejection fraction was 61% by gated SPECT.

D – Polar maps (at 6-month follow-up): SSS – 8, SRS – 0, SDS – 8. MPI - myocardial perfusion imaging, SPECT - single-photon emission computed tomography.

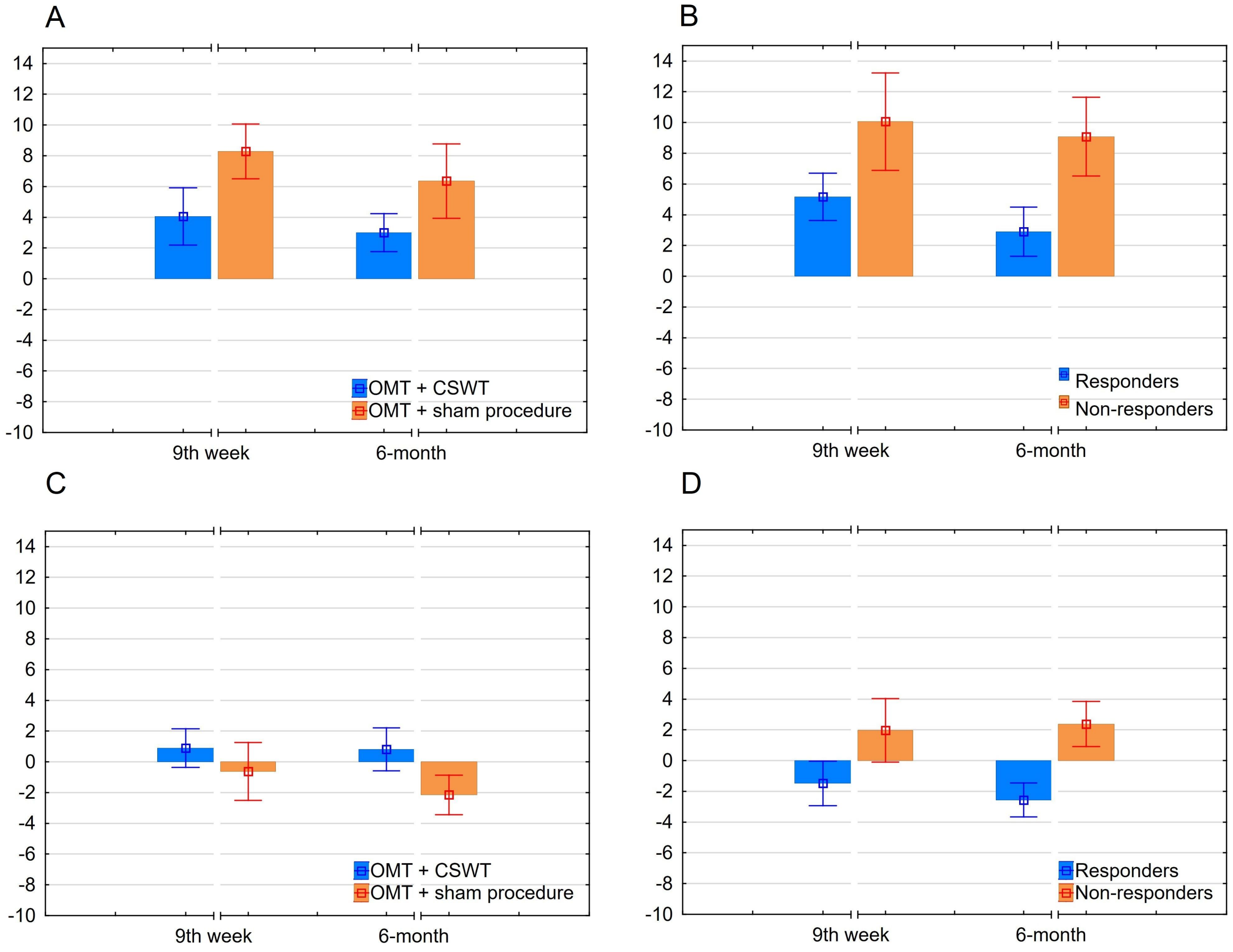

Figure S2. Mean percentage change of catestatin and endocan levels in sub-study groups.
Mean percentage change of catestatin levels: A - study groups. Mean percentage change of catestatin levels: B - in sub-groups. Mean percentage change of endocan levels: C - study groups. Mean percentage change of endocan levels: D - in sub-groups.
Data are shown as mean ± standard error of the mean (SEM). Percentage change = [(follow up time point value - baseline value)/ baseline value]x100.
